# *Paeonia lactiflora* Extract Attenuating Cerebral Ischemia and Arterial Intimal Hyperplasia Is Mediated by Paeoniflorin via Modulation of VSMC Migration and Ras/MEK/ERK Signaling Pathway

**DOI:** 10.1155/2013/482428

**Published:** 2013-06-02

**Authors:** Yuh-Fung Chen, Kuo-Jen Wu, W. Gibson Wood

**Affiliations:** ^1^Department of Pharmacology, China Medical University, No. 91 Hsueh-Shih Road, Taichung 40402, Taiwan; ^2^Department of Pharmacy, China Medical University Hospital, No. 2 Yu-Der Road, Taichung 40447, Taiwan; ^3^Department of Pharmacology, University of Minnesota and Geriatric Research, Education and Clinical Center, VA Medical Center, Minneapolis, MN 55455, USA

## Abstract

*Paeonia lactiflora* is a well-known traditional Chinese medicine. Paeoniflorin is an active component found in *Paeonia lactiflora*, which is used to treat smooth muscle spasms and pain and to protect the cardiovascular system. The objective of this study was to determine if *Paeonia lactiflora* would be protective in rodent models of cerebral ischemia and arterial intimal hyperplasia. *Paeonia lactiflora* extract (PLex) and paeoniflorin (PF) significantly attenuated cerebral infarction in ischemia/reperfusion injury rats and the severity of intimal hyperplasia in mice where the carotid artery was ligated. PLex and PF reduced PDGF-stimulated VSMC proliferation and migration in a dose-dependent manner by MTT, wound healing, and transwell assays. PF significantly reduced protein levels of Ras, MEK, p-MEK and p-ERK, but not MMP-2 and MMP-9. In summary, *Paeonia lactiflora* reduced cerebral ischemia and arterial intimal hyperplasia which were mainly made via the intermediary of PF. The protective effect of PF was related to the modulation of the Ras/MEK/ERK signaling pathway.

## 1. Introduction

Cerebral ischemia and infarction are most commonly associated with atherosclerotic disease and stroke in the carotid and vertebrobasilar circulatory systems [1]. Cerebral infarction and resulting pathology are a leading cause of disability and mortality. Restoration of blood flow is essential to prevent irreversible ischemic tissue injury. However, reperfusion can have local and systemic inflammatory effects that enhance tissue injury [2]. Abnormal proliferation and migration of VSMCs in arterial injury can lead to intimal hyperplasia [3–6]. The inability to limit intimal hyperplasia most likely relates to its complex nature, which involves inflammatory cells and their mediators, angiogenesis, and vascular smooth muscle cells (VSMC) growth and migration. There is a close relationship between vascular intimal hyperplasia and atherosclerosis, venous thrombosis, and balloon injury, which are a result of abnormal VSMC proliferation and migration [7, 8]. Integrity of VSMC is required for optimal vascular function [9]. When the vascular endothelium is damaged, platelet-derived growth factor (PDGF) is released. PDGF stimulates cell growth and also promotes migration in VSMC and causes the development of intimal hyperplasia and restenosis [10–12]. Over the past decade, possible reperfusion injury has advanced understanding of pathophysiology. Cerebrovascular diseases can only be controlled by medication or surgery, but frequently patients relapse months or years later [13, 14]. Therefore, discovering drugs that effectively attenuate ischemia-reperfusion (I/R) injury is a high priority.

Drugs that inhibit the activation of PDGF and VSMC proliferation and migration could be protective. *Paeonia lactiflora* Pall. (Ranunculaceae) is a well-known traditional Chinese herbal medicine which is used for relaxing abdominal spasm, relieving pain, and improving blood circulation. *Paeonia lactiflora* has been used as an antiallergic, anticonvulsant, anti-inflammatory, and antispasmodic herbal medicine. Paeoniflorin, a major component of *Paeonia lactiflora* has potent antispasmodic, anticoagulative, analgesic, and anti-inflammatory activities [15–17]. The biological effects of *Paeonia lactiflora* and PF on carotid vascular smooth muscle cells have not been explored. The objective of this study was to evaluate the effects of* Paeonia lactiflora* extract and paeoniflorin on cerebral ischemia/reperfusion injury and carotid-ligation artery *in vivo*, and PDGF-BB-stimulated vascular smooth cell (A7r5) migration *in vitro*. 

## 2. Materials and Methods

### 2.1. Preparation of Plant Extract

**Figure 1:**
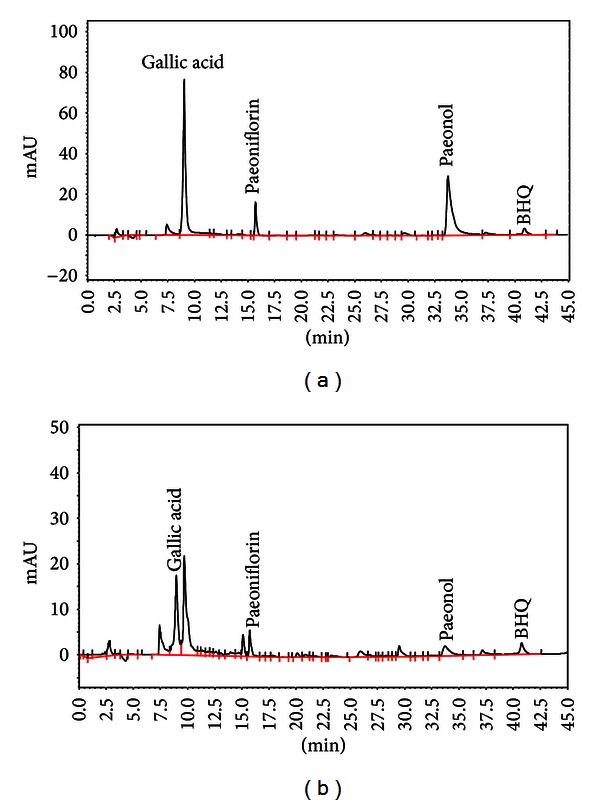
HPLC chromatograms of *Paeonia lactiflora* at 250 nm. Trace (a) Standard, gallic acid (50 *μ*g/mL), paeoniflorin (100 *μ*g/mL), and paeonol (50 *μ*g/mL). (b) PLex (2.5 mg/mL) The content of paeoniflorin in PLex is 9.94 *μ*g/mg. BHQ: tert-butylhydroquinone as an internal standard.

*Paeonia lactiflora* Pall. was obtained from the Department of Pharmacy, China Medical University Hospital, Taiwan. Two kilograms of *Paeonia lactiflora* were extracted by using water (100°C) for a 2 hr cycle two times. The extract of *Paeonia lactiflora* (abbreviated as PLex) was collected, filtered, and frozen to a dry powder, and the yield was 8.23% of the total extract. PLex was freshly prepared in distilled water before experiments. HPLC-DAD (diode array detector) was used for the study of paeoniflorin (abbreviated as PF) in PLex. The HPLC-DAD conditions were based on an earlier report [18]. An HPLC chromatogram of PLex and PF is shown in Figure 1. The content of PF is 9.94 *μ*g/mL in 2.5 mg/mL PLex.

### 2.2. Cell Line

A VSMC line, A7r5, was purchased from Bioresource Collection and Research Center, Hsinchu, Taiwan. A7r5 cells were plated onto 6-well plates in DMEM, supplemented with 10% FBS, 100 units/mL penicillin, 100 *μ*g/mL streptomycin, and 2 mM L-glutamine, and grown at 37°C under a humidified 5% CO_2_ and 95% air at one atmosphere. 

### 2.3. Chemicals

Chemicals were purchased from the following companies. PDGF-BB (PDGF) was from Sigma (St. Louis, MO, USA). Zoletil was from Virbac Laboratories (Carros, France). Anti-MMP-2, anti-MMP-9, anti-Ras, anti-MEK, anti-phosphor-MEK1/2, anti-ERK, and anti-phospho-ERK1/2 antibodies were from Abcam (Cambridge, UK). Anti-*β*-actin was from Santa Cruz Biotechnology (Santa Cruz, CA, USA). DMEM, FBS, penicillin/streptomycin, and glutamine were from Thermo Scientific Inc. (Waltham, PA, USA). 2,3,5-Triphenyltetrazolium chloride (TTC) was from Sigma-Aldrich (St. Louis, MO, USA). Paeoniflorin (PF) was purchased from IvyChemical Company (Cherry Hill, NJ, USA).

### 2.4. Animals

Male Sprague-Dawley (SD) rats, weighing 275–300 g, and male ICR mice, weighing 25–30 g, were purchased from BioLASCO Co., Ltd. (Taipei City, Taiwan). All animals were fed with standard chow and housed in standard cages at a constant temperature of 22 ± 1°C. Relative humidity 55% ± 5% with 12 hr inverted light-dark cycle for 1 week at least before the experiment. The experimental protocol was approved by the Committee on Animal Research, China Medical University (permit number: 101-251). All surgery was performed under zoletil anesthesia and all efforts were made to minimize suffering. The minimum number of animal required to obtain consistent data was used. Five to six animals were used in each group.

### 2.5. Surgical Procedures of Cerebral Ischemia/Reperfusion

Male SD rats were deeply anesthetized by intraperitoneal injection of 25 mg/kg of zoletil. The animal model of surgical cerebral infarction and ischemia/reperfusion were modified as described by Wu et al. [18]. Each rat was placed supine, and both common carotid arteries were exposed through a midline incision in the neck. Then, both common carotid arteries were tied off with plastic line (0.1 mm in diameter). Distilled water and different concentrations of PLex (50, 100, 200 mg/kg) and PF (0.5, 1.0, 2.0 mg/kg) were orally administered 60 min before 3-vessels occlusion in rats, respectively. After 90 min occlusion followed by reperfusion for 24 hr, the brain of each rat was removed after transcardiac perfusion of 0.9% NaCl. Each brain was then placed into a plastic rat brain matrix and was sectioned coronally into 2 mm slices. The slices were stained with 2% 2,3,5-triphenyltetrazolium (TTC) solution at room temperature for 15 min. The slices were fixed with 10% formalin solution. The cerebral infarction areas of the first six sections from the frontal lobe were measured using an image-analysis system (Image-Pro Plus 6.0 Media Cybernetics, USA). The ratio of infarction area to total brain area in each section of the rat brain was calculated, and the data were expressed as a percentage (%).

### 2.6. Animal Carotid Ligation Model

The carotid ligation model was modified from a previous report [19]. Male ICR mice weighing 25 to 30 g were used for the carotid-ligation model. All surgical procedures were performed with animals under general anesthesia (zoletil 25 mg/kg, i.p.) using sterile surgical techniques with a dissecting microscope. A midline neck incision was used to expose the left common carotid artery. The artery was completely ligated just proximal to the carotid bifurcation. The right carotid artery served as a noninjured control. After ligation, the incision was closed, and the animals were allowed to recover. On the day of carotid ligation, mice were randomized into 3 treatment groups and they were anesthetized as described above. Group 1 was treated with distilled water and groups 2 and 3 were treated with PLex (100 and 200 mg/kg p.o.). PLex and distilled water were given by gastric gavages on the second day after carotid ligation once a day for 28 consecutive days. All animals were euthanized on day 29 after ligation for histomorphometric analysis. The left and right common carotid arteries were harvested, dehydrated in ethanol and xylene, and embedded in paraffin. Haematoxylin-eosin staining and PCNA antibody staining were used for the carotid arteries [19]. Arterial sections (2.5 *μ*m) were selected and stained by use of a haematoxylin-eosin solution. Images were digitized and analyzed with Image-Pro software. The areas of the lumen, internal elastic lamina (IEL), and external elastic lamina (EEL) were determined by computerized planimetry. The luminal area, intimal area, medial area, and intimal/medial ratios were calculated. The intimal area was calculated by subtracting the luminal from the IEL area, and medial area was determined by subtracting the IEL area from the EEL area [19]. The ratio of intimal to medial area (I/M ratio) was calculated and analyzed. Proliferating cell nuclear antigen (PCNA) was also determined in arterial sections [20].

### 2.7. Cell Proliferation Assay

A7r5 proliferation was measured by determining cell number. The cells (1 × 10^4^ cells/well) were seeded onto 12 well plates and grown in DMEM containing 10% FBS for 24 hr. The cells were then cultured with medium containing PDGF (30 ngmL) and PLex (50, 100, and 200 *μ*g/mL) or PF (5, 10, and 20 *μ*M) for 24 to 72 hr. MTT (200 *μ*L, 0.5 mg/mL) was added to each well and incubated for 4 hr. Five hundred *μ*L of DMSO was added to each well to solubilize the formed formazan crystals. The optical density was measured at 570 nm with a spectrophotometer (BioRad Laboratories, Hercules, CA, USA).

### 2.8. *In Vitro* Wound Healing Assay

To evaluate the impact of PLex (50, 100, and 200 *μ*g/mL) and PF (5, 10, and 20 *μ*M) on VSMC migration, a wound healing assay was used. A7r5 cells (2 × 10^5^ cells/well) were plated in 6-well plates, and the wound was induced with a single scratch using a sterile pipette tip. Cells were then incubated with or without PDGF (30 ng/mL), PLex (50, 100, and 200 *μ*g/mL) and PF (5, 10, and 20 *μ*M) in serum-reduced DMEM (containing 0.5% fetal bovine serum). The rate of wound closure was determined by photographing cells using a phase-contrast microscope at 24, 48, and 72 hr. Cell migration was expressed as the migration distance of drug-treated cells (mm) divided by the migration distance of untreated cells (mm).

### 2.9. Transwell Migration Study

The effects of PLex and PF on VSMC migration were further investigated using a transwell migration chamber with a collagen-coated polycarbonate filter. A7r5 cells (5 × 10^5^ cells/well) were incubated on the transwell apparatus (a 6.5-mm polyethylene terephthalate membrane with 8-*μ*m pores; Millicell, Millipore Inc, Billerica, MA01821, USA) and treated with PDGF (30 ng) and PLex (50, 100, and 200 *μ*g/mL) or PF (5, 10, and 20 *μ*M) for 48 hr. The cells were then trypsinized, resuspended in 0.5% FBS medium. FBS/DMEM (10%) was added to the bottom chamber of each well as the chemoattractant. Cells were allowed to migrate through the membrane to the underside of the apparatus for 8 hr and were then fixed with methanol for 10 min and stained with Giemsa solution for 30 min. The cells migrating to the lower outside of the insert membrane were counted manually under a microscope using the NIS-Elements software (Nikon Inc, Melville, NY, USA). 

### 2.10. Protein Preparation and Western Blot Analysis

A7r5 cells (5 × 10^6^ cells) were seeded in 10 cm dish and treated with PF (5, 10, and 20 *μ*M) for 4 hr for phosphor-protein expression and for 48 hr for protein expression. The cells were harvested and washed with cold 1X PBS. The total protein concentration was determined using a BCA assay kit (Pierce Biotechnology Inc., Rockford, IL, USA). Equal amounts of cell lysate were run on 10%–12% SDS-polyacrylamide gel electrophoresis (SDS-PAGE) and electrotransferred to polyvinylidene fluoride membranes (PVDF, Thermo Scientific Inc, Waltham, PA, USA) using iBotTM Dry Blotting System (Invitrogen). The blot was soaked in blocking buffer (5% nonfat dry milk/0.05% Tween 20 in 20 mM TBS at pH 7.6) at room temperature for 1 hr and then incubated with anti-MMP-2, anti-MMP-9, anti-Ras, anti-ERK, anti-phospho-ERK1/2, anti-MEK, and anti-phosphor-MEK1/2 and *β*-actin antibodies in blocking buffer, respectively, at 4°C overnight. *β*-Actin was used as an internal loading control. Membranes were washed with Tris-buffered saline/Tween 20 three times for 10 min and then incubated with secondary horseradish peroxidase (HRP-) conjugated antibody. The blots were developed using a chemiluminescence (ECL) detection kit (Millipore, Billerica, MA, USA) followed by development on Kodak BioMax MR film (Eastman Kodak, Rochester, NY, USA). All results are from 6 independent experiments.

### 2.11. Statistical Analysis

The data are represented as mean ± SE. Groups were compared by one-way analysis of variance (ANOVA) followed by Scheffe's test. A *P* value  <0.05 was considered statistically significant.

## 3. Results

### 3.1. Effects of PLex and PF on Cerebral Infarct Volume

**Figure 2:**
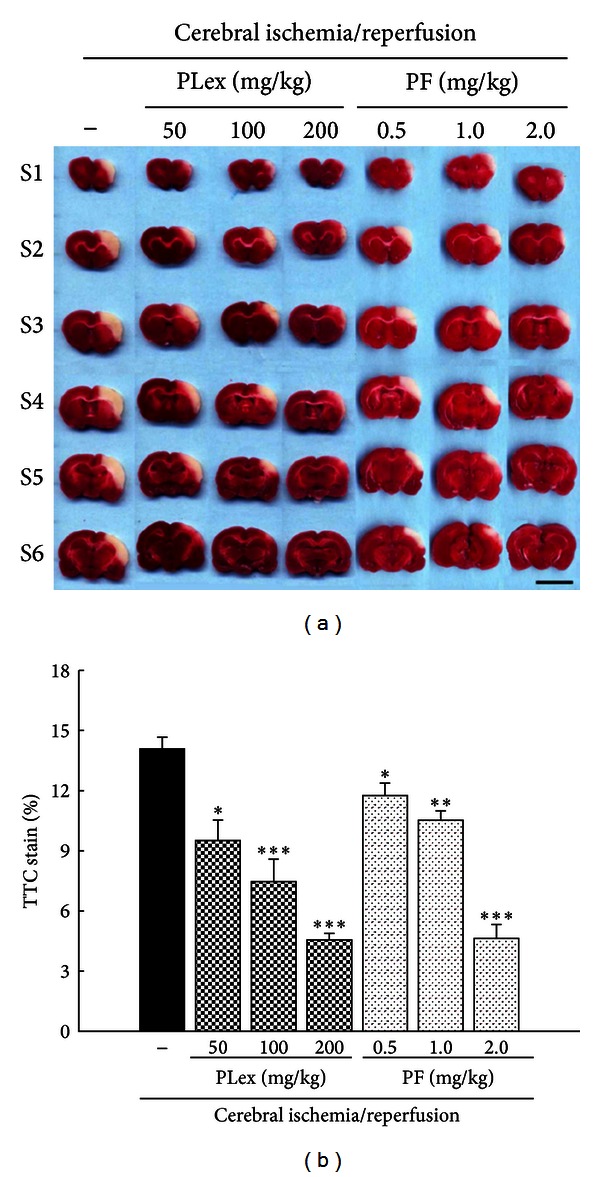
Effects of PLex and PF on cerebral infarction induced by ischemia/reperfusion injury. Coronal section of brain after ischemia for 90 min and followed by reperfusion for 24 h. Then staining with TTC, the infarction areas appeared white and noninfarction areas appeared red-purple in color. S1→S6: slices from frontal lobe. (a) Treatment with PLex and PF. (b) Statistic percentage of cerebral infarction area of PLex and PF. Scale bar = 1 cm. *P < 0.05, **P < 0.01, and **P < 0.001 compared with the control group (*n* = 6).

Blood flow was blocked for 90 min in both common carotid arteries and the right cerebral artery and then reperfused for 24 hr. After staining with TTC, the infarction areas were visibly white, and noninfarction areas were red-purple in color. PLex and PF significantly decreased the infarct volume in a dose-dependent manner (Figure 2(a)). PLex (50, 100, and 200 mg/kg) reduced the infarct area by 32.49%, 47.00%, and 67.63%. The inhibitory percentages of PF (0.5, 1.0, and 2.0 mg/kg) on the infarct area were 16.51%, 25.27%, and 67.08% as shown in Figure 2(b), respectively. 

### 3.2. Effect of PLex on Intimal Hyperplasia

**Figure 3:**
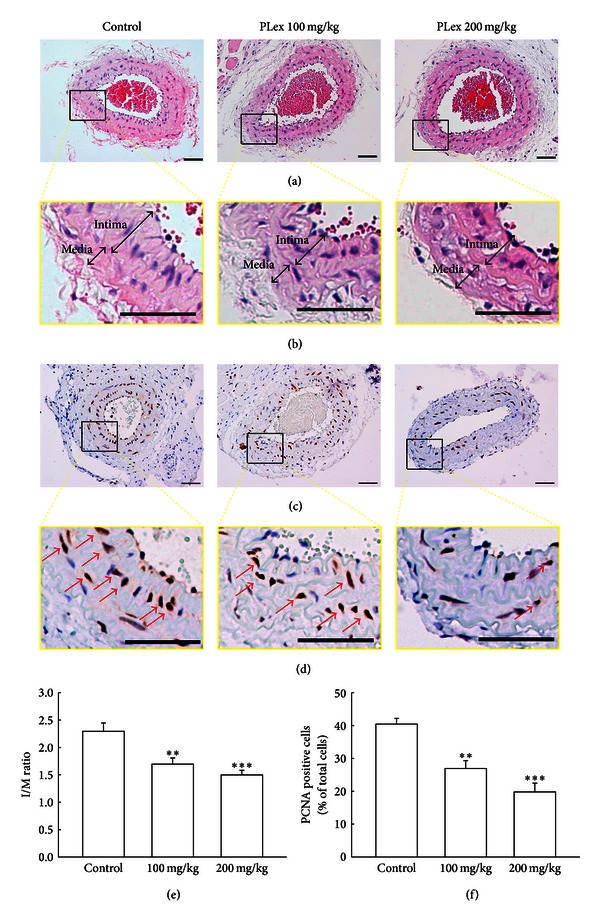
Effects of PLex on carotid-ligation induced intimal hyperplasia in mice. Different doses of PLex (100 and 200 mg/kg) were orally administered once a day for 28 days and the sham group received normal distilled water. (a) and (b) represent photomicrographs of hematoxylin-eosin staining of arterial sections 28 days after carotid ligation, and (c) and (d) represent PCNA-immunoreactivity staining of arterial sections (200x). (e) Severity of intimal hyperplasia was calculated according to intima/media area (I/M) ratio. (f) Percentage of PCNA positive cells per total cells. The arrow was indicating the PCNA-positive cell. Scale bar = 50 *μ*m. **P < 0.01 and ***P < 0.001 compared with the control group (*n* = 5).

After carotid ligation, mice were treated with PLex (100 and 200 mg/kg) or distilled water (control group) for 4 weeks. The carotid artery, absolute lumen area, intimal area, medial area, and intima/media (I/M) ratios were determined. The morphology of the intima/medial area was abnormal after carotid ligation for 4 weeks (I/M ratio = 2.29 ± 0.15). The PLex-treated mice significantly improved intimal hyperplasia after carotid ligation compared with the control group. I/M ratio was 1.69 ± 0.11 (100 mg/kg) and 1.50 ± 0.08 (200 mg/kg) (Figures 3(a) and 3(b)). The average inhibitory rate of PLex was 26.14% (100 mg/kg) and 34.67% (200 mg/kg) compared with the control group (Figure 3(e)). Arterial sections stained to detect the proliferating cell nuclear antigen (PCNA) are shown in Figures 3(c) and 3(d). The percentages of PCNA positive cells per total cells were 40.44 ± 1.75% in the control group, and 26.92% ± 2.40% and 19.79% ± 2.69% in PLex (100 and 200 mg/kg) treated groups, respectively. The average inhibitory rate of PLex on PCNA was 33.43% (100 mg/kg) and 51.06% (200 mg/kg) compared with the control group (Figure 3(f)).

### 3.3. Effects of PLex and PF on VSMC Proliferation

**Figure 4:**
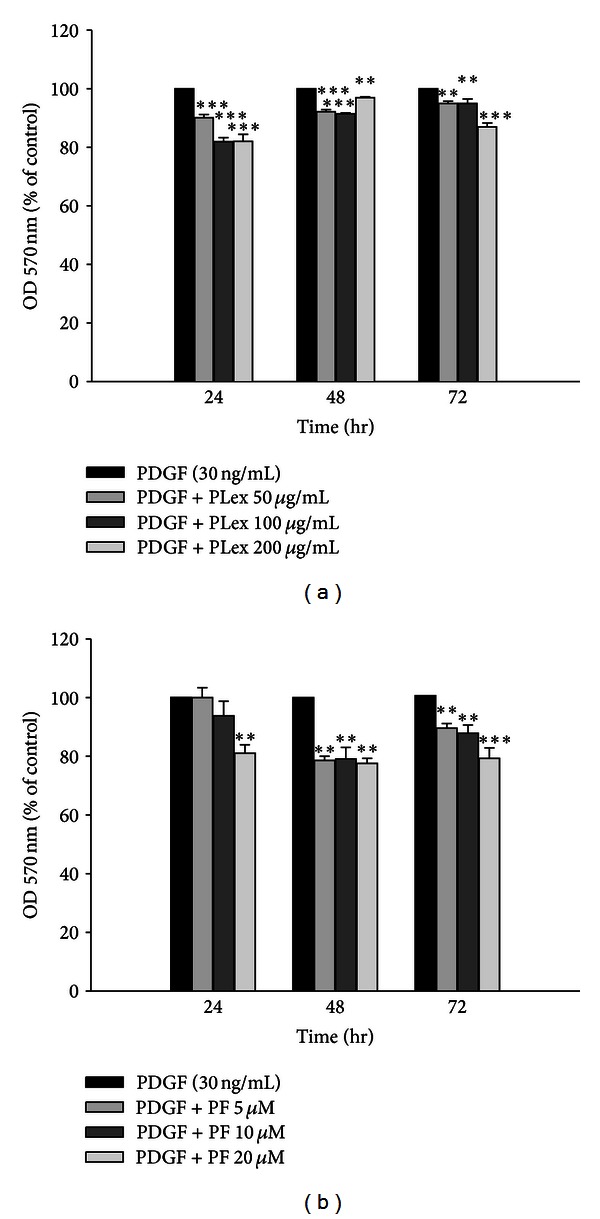
Effects of PLex and PF on PDGF-induced VSMC proliferation by MTT assay. PLex (a) and PF (b) inhibit vascular smooth cell proliferation in response to PDGF in a dose-dependent and a time-dependent manner. Statistical difference in 24, 48, and 72 hr at different concentrations of PLex (a) and PF (b). **P < 0.01, ***P < 0.001 compared with PDGF control group (*n* = 10).

To investigate the effects of PLex and PF on PDGF-stimulated VSMC proliferation, the cells were exposed to 50–200 *μ*g/mL of PLex or 5–20 *μ*M PF for 24–72 hr.  Figure 4(a) shows that 50–200 *μ*g/mL PLex inhibited PDGF-stimulated VSMC proliferation. The inhibitory percentages of PLex (200 *μ*g/mL) were 18%, 3.05% and 13.08% at 24, 48, and 72 hr, respectively.  Figure 4(b) showed that the inhibitory percentages of PF (5~20 *μ*M) were 18.98%, 22.45%, and 20.75% at 24, 48, and 72 hr, respectively.

### 3.4. Effect of PLex and PF on VSMC Migration

**Figure 5:**
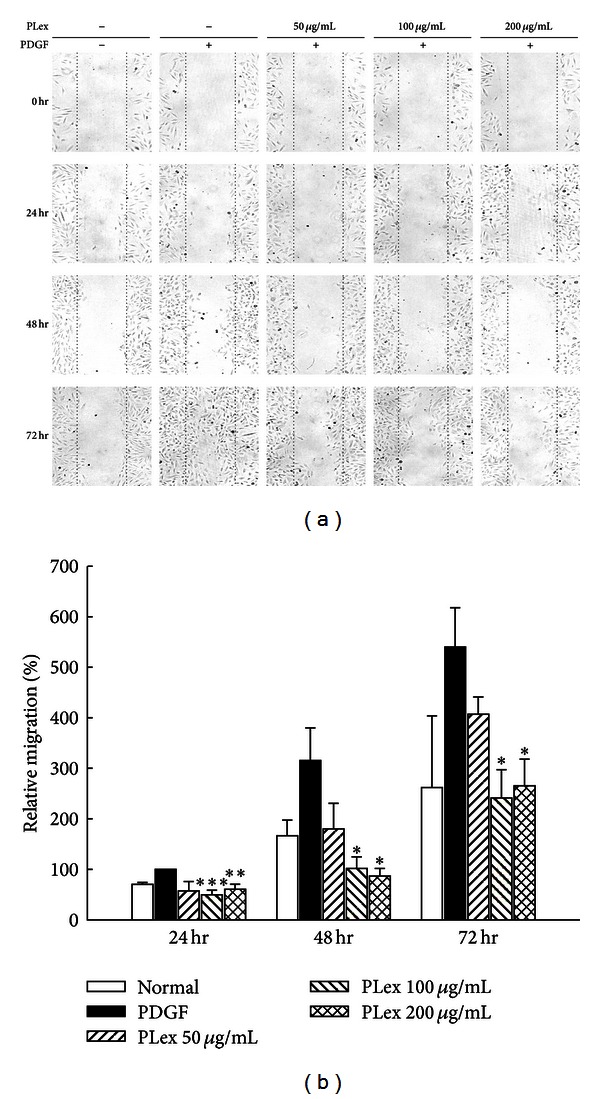
Effects of PLex on PDGF-induced VSMC migration by wound healing assay. (a) A typical trace of PLex (5, 10, and 20 *μ*g/mL) inhibited in response to PDGF-induced VSMC migration. (b) Statistical differences in 24, 48, and 72 hr at different PLex concentrations, respectively. Normal group was treated with vehicle solution. *P < 0.05, **P < 0.01, and ***P < 0.001 compared with PDGF control group (*n* = 6).

**Figure 6:**
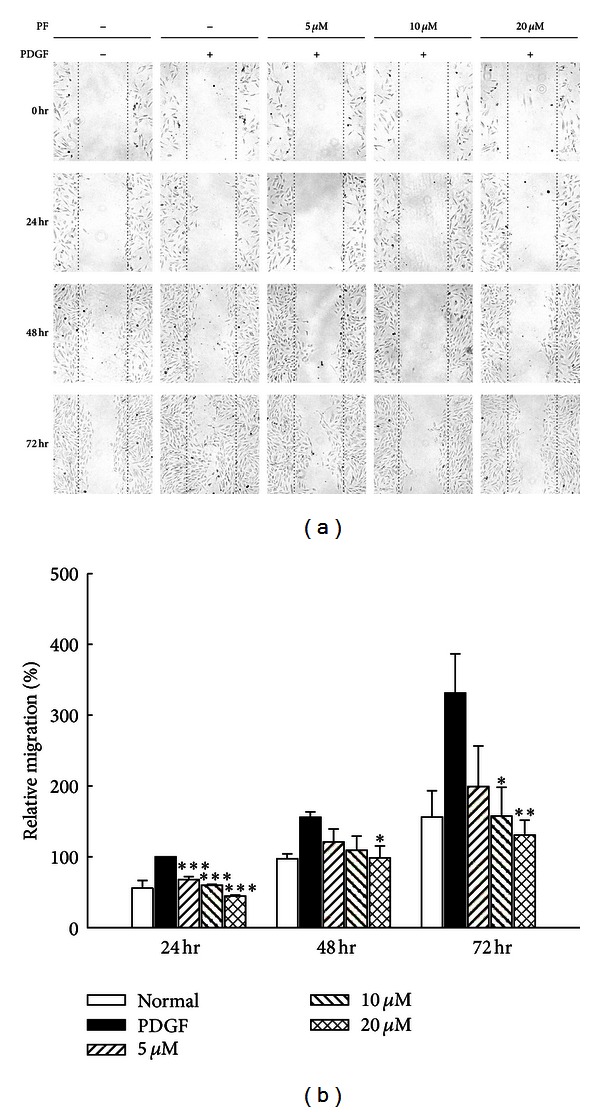
Effects of PF on PDGF-induced VSMC migration by wound healing assay. (a) A typical trace of PF (5, 10, and 15 *μ*M) inhibited in response to PDGF-induced VSMC migration. (b) Statistical differences in 24, 48, and 72 hr at different PF concentrations, respectively. Normal group was treated with vehicle solution. *P < 0.05, **P < 0.01, and ***P < 0.001 compared with PDGF control group (*n* = 6).

**Figure 7:**
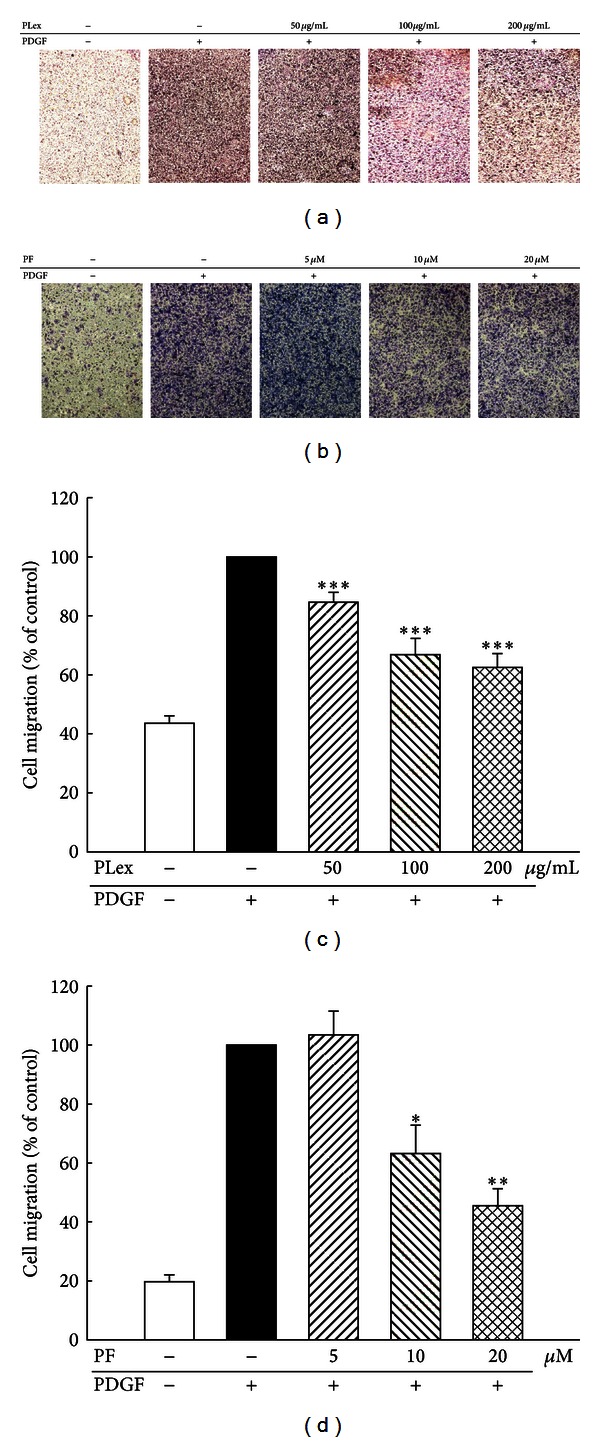
Effects of PLex and PF on PDGF-induced VSMC migration by transwell assay. PLex (a) and PF (b) inhibit vascular smooth cells migration in response to PDGF in a dose-dependent and a time-dependent manner. Statistical differences of PLex (c) and PF (d) inhibit vascular smooth cells migration in response to PDGF, ***P < 0.001 compared with PDGF treated group (*n* = 6).

The effects of PLex and PF on VSMC migration were evaluated by wound-healing at 24, 48, and 72 hr after treatment. VSMCs were pretreated with or without PLex (50, 100, and 200 *μ*g/mL) and PF (5 *μ*M, 10 *μ*M, and 20 *μ*M), then stimulated with PDGF (30 ng/mL) (Figures 5(a) and 6(a)). The migrated cell numbers induced by PDGF across the membrane were significantly inhibited by both PLex (Figure 5(b)) and PF (Figure 6(b)). The effects of PLex and PF on VSMC migration were further confirmed by a modified Boyden chamber experiment. VSMC numbers were reduced by pretreatment with PLex (Figure 7(a)) and PF (Figure 7(b)). The inhibitory percentages were 13.16%, 34.01%, and 37.87% by PLex (50, 100, and 200 *μ*g/mL) (Figure 7(c)) and 36.74% and 54.42% by PF (10 and 20 *μ*M) (Figure 7(d)) compared with a positive control group treated with PDGF, respectively.

### 3.5. Effects of PF on MMPs and Ras/MEK/ERK Protein Levels

**Figure 8:**
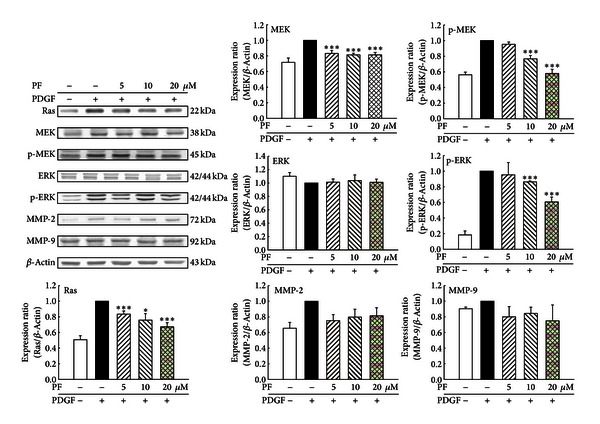
Effects of PF on Ras/MEK/ERK protein expression in PDGF-induced VSMCs. The protein expressions were determined by western blot analysis. Ras, MEK, p-MEK, and p-ERK were markedly inhibited in a dose-dependent manner. *P < 0.05 and ***P < 0.001 compared with PDGF control group (*n* = 6).

Protein levels of VSMC were examined by western blotting. Various concentrations of PF significantly reduced protein abundance of Ras, MEK, p-MEK, and p-ERK (Figure 8). PF reduced the expressions of MMP-2 and MMP-9. However, there is no statistical significance. Percentages reduction of PF (5, 10, and 15 *μ*M) on protein levels were as follows: Ras were 16.5%, 24.25%, and 32.75%, respectively; MEK were 16.8%, 18.6%, and 18.6%, respectively; p-MEK were 4.67%, 23.33%, and 42.00%, respectively; p-ERK were 4.67%, 13.67%, and 39.33%, respectively.

## 4. Discussion

Cerebral ischemia induces hypoxia which can cause brain tissue damage and reperfusion-induced reoxygenation often exacerbates tissue injury and inflammation [21]. Inflammation is a major contributor to stroke-related brain injury. Individual components of the inflammatory cascade may have detrimental or beneficial effects depending on the stage of tissue injury, the magnitude of the response, and whether these components stimulate neuroprotective pathways [22–24].

Traditional Chinese herbal medicine has a long history as a treatment for conditions associated with stroke and recent reports support such an approach [25]. We found that administration of PLex and PF significantly reduced cerebral infarction size, which may be acting through PF. PF has a characteristic monoterpene glucoside which has an anti-inflammatory effect on the cerebral infarction [17, 26]. There have been no reports on the anticerebral ischemic effect of *Paeonia lactiflora*. Only the protective effects of Guizhi-Fuling-Capsules (GZFLC) on rat brain ischemia/reperfusion injury were reported [27]. It is suggested that the protective effect of GZFLC on rat brain ischemia-reperfusion injury is partly due to its inhibition of proinflammatory cytokines IL-1*β* and TNF*α* and upregulated expressions of anti-inflammatory cytokines IL-10 [27]. *Paeonia lactiflora* is one of the component herbal of GZFLC. PF, a component in PLex, inhibits NF*κ*B expression in chronic hypoperfusion rat and has anti-inflammatory properties. PF reduces cerebral infarction area by inhibiting NF*κ*B, IL-1*β*, and TNF*α* [17]. Data from this study revealed that PLex inhibited intimal hyperplasia via the action of PF on Ras/MEK/ERK signaling pathway modulation.

Blood vessels in response to injury or atherosclerosis can develop intimal thickening, vessel occlusion, and cerebral infarction [14, 28, 29]. PDGF, which is produced by VSMC, vascular endothelial cells, platelets, or macrophages, stimulates VSMC proliferation and migration. PDGF acts via mitogen-activated protein kinase and it plays a pivotal role in intimal hyperplasia in the injured vascular walls [14, 28, 29]. PDGF receptors were markedly upregulated after injury to the vessel wall [30], and an antibody to PDGF inhibited the neointimal smooth muscle accumulation after angioplasty [31]. PDGF activates the tyrosine kinase receptor and triggers a cascade of tyrosine phosphorylations that lead to the formation of a ternary complex connecting the receptor to ras. Ras proteins are active transducers of mitogenic signals from the plasma membrane to the nucleus in many cell types. Active ras binds the serine protein kinase raf, which activates MEK/ERK signaling cascade to regulate gene expression. It has been reported that modulation of VSMC growth has critical therapeutic implications [32]. MEK (MAPK; mitogen-activated protein kinase) pathways play an important role in promoting VSMC proliferation [33] and are activated by vascular injury. The MAPK/ERK pathway is a series of protein reactions in cells which are phosphorylated during PDGF-stimulated cell migration [34]. This cascade culminates in the activation of nuclear transcription factors [35]. Most peptidic growth factors (GFs) that act as mitogens for different cell types bind to tyrosine kinase receptors and trigger complex intracellular signal transduction pathways leading to cell proliferation. Among the early events induced by GFs, cytosolic calcium increase plays a key role [36]. Elevation of intracellular calcium by ionomycin has been reported to increase migration of vascular smooth muscle cells [37]. Previous reports indicated that PF antagonizes veratrine-induced isolated rat aorta contraction which may be mediated by blocking intracellular calcium [38]. Paeoniae rubra relaxes vascular smooth muscle via inhibition of L-type Ca^2+^ channels [39]. In the present study, PF attenuated the PDGF-stimulated Ras expression in a dose-dependent manner possibly by inhibiting Ca^2+^ channels.

When activated MAPK (p-ERK) translocates into the nucleus, multiple cytoplasmic matrix and transcription factors (matrix metalloproteinases; MMPs) are phosphorylated. MMPs regulate migration, proliferation, and death of vascular smooth muscle cells by degrading matrix and nonmatrix substrates [40]. MMP plays a vital role in the degradation of the extracellular matrix that facilitates VSMC migration. During the process of atherosclerosis and neointimal formation, various cytokines and growth factors stimulate VSMC to express MMP [7]. MMP-2 and MMP-9 are necessary for the migration of many cell types [41]. Inhibition of the activated ERK pathway by drugs or gene therapy can reduce neointimal hyperplasia [42]. Inhibition of PDGF-stimulated VSMC proliferation and migration can attenuate the development of intimal hyperplasia, which may have therapeutic potential in the prevention or treatment of cerebrovascular diseases [32]. 

In conclusion, results of the present study indicate that *Paeonia lactiflora* ameliorates cerebral ischemia and arterial intimal hyperplasia which is mediated by paeoniflorin via modulation of Ras/MEK/ERK pathway of vascular smooth muscle cells.
